# Efficacy and safety of Telitacicept in patients with IgA nephropathy

**DOI:** 10.12669/pjms.39.6.8393

**Published:** 2023

**Authors:** Dongrui Liang, Xiaodong Li

**Affiliations:** 1 Dongrui Liang, Department of Ophthalmology, Baoding No. 1 Central Hospital of Hebei Medical University, Baoding, Hebei, China; 2 Xiaodong Li Department of Nephrology, Baoding No. 1 Central Hospital of Hebei Medical University, Baoding, Hebei, China

Chronic kidney disease has become a worldwide public health health.^1^ Based on a nationwide cross-sectional survey conducted during the period of 2018-2019, it was found that the incidence of chronic kidney disease in China stands at 8.2%, impacting a staggering 82 million individuals.^2^ While the prevalence of metabolically associated secondary kidney diseases, such as diabetes and hypertension, is on the rise, it is noteworthy that the proportion of IgA nephropathycontinues to remain substantial. As the pathogenesis of autoimmune nephropathy is being extensively investigated and biomedical research advances at a rapid pace, the utilization of monoclonal antibodies, such as rituximab and belimumab, has witnessed a growing application in the field of kidney disease. Telitacicept, being a novel biological agent, has exhibited promising therapeutic efficacy and safety in preliminary clinical studies conducted within the realm of IgA nephropathy.^3^

B lymphocytes assume a pivotal role in both the onset and advancement of IgA nephropathy. These cells possess the ability to undergo differentiation into plasma cells, which subsequently release auto-antibodies. These auto-antibodies, whether targeting specific or non-specific kidney antigens, contribute to the formation of immune complexes that ultimately induce renal damage. The development and differentiation of B cells involve the participation of various cytokines, among which BAFF (B-cell activating factor) and APRIL (a proliferation-inducing ligand) hold paramount significance as key factors.^3^ Xin et al conducted a study which divulged that IgA nephropathy patients exhibited elevated serum levels of BAFF, which were found to be linked to both clinical manifestations and pathological characteristics of the disease.^4^ Furthermore, they observed an up-regulation in the expression of the APRIL gene within the tonsil germatogenesis center of IgA nephropathy patients, and this was found to correlate with serum levels of Gd-IgA1 and the severity of the disease.^5^ These findings indicate that both BAFF and APRIL may play a crucial role in the generation of Gd-IgA1.^3^ Consequently, the targeting of BAFF and APRIL presents itself as a promising therapeutic strategy for IgA nephropathy.

Telitacicept, with its indication for IgA nephropathy, received approval from the United States Food and Drug Administration (FDA). Notably, the Phase I clinical trial was exempted, and the Phase II clinical trial was directly conducted. Subsequently, on November 18, 2022, the FDA granted approval for a Phase III clinical trial of Telitacicept in the United States specifically for the treatment of IgA nephropathy. Consequently, Telitacicept has demonstrated the ability to reduce proteinuria in high-risk patients with IgA nephropathy, thereby potentially mitigating the risk of disease progression.^5^ Nevertheless, it is important to note that further basic and clinical trials are necessary to validate these findings. Additionally, the identification of suitable candidate individuals for targeted treatment, based on factors such as abnormal expression of BAFF/APRIL, remains a challenge that needs to be addressed prior to the widespread its application, with the aim of maximizing the potential benefits for patients.^6^ In summary, Taitasip exhibits promising prospects for its application in IgA nephropathy, while also providing novel insights and directions for the clinical management of other autoimmune renal diseases.

## Authors Contributions:

**XL:** Design and revision. **DL:** Writing and literature review. All authors contributed to the article and approved the submitted version.
